# Estimating Kidney Function in HIV-Infected Adults in Kenya: Comparison to a Direct Measure of Glomerular Filtration Rate by Iohexol Clearance

**DOI:** 10.1371/journal.pone.0069601

**Published:** 2013-08-08

**Authors:** Christina M. Wyatt, George J. Schwartz, Willis Owino Ong'or, Joseph Abuya, Alison G. Abraham, Charles Mboku, Loice B. M'mene, Winnie J. Koima, Mathew Hotta, Paula Maier, Paul E. Klotman, Kara Wools-Kaloustian

**Affiliations:** 1 Department of Medicine, Division of Nephrology, Mount Sinai School of Medicine, New York, New York, United States of America; 2 Department of Pediatrics, Division of Nephrology, University of Rochester Medical Center, Rochester, New York, United States of America; 3 School of Medicine, College of Health Sciences, Moi University, Eldoret, Kenya; 4 Department of Epidemiology, Johns Hopkins Bloomberg School of Public Health, Baltimore, Maryland, United States of America; 5 Academic Model Providing Access to Healthcare (AMPATH), Eldoret, Kenya; 6 Department of Medicine, Baylor College of Medicine, Houston, Texas, United States of America; 7 Department of Medicine, Division of Infectious Diseases, Indiana University School of Medicine, Indianapolis, Indiana, United States of America; Lausanne University Hospital and University of Lausanne, Switzerland

## Abstract

**Background:**

More than two-thirds of the world's HIV-positive individuals live in sub-Saharan Africa, where genetic susceptibility to kidney disease is high and resources for kidney disease screening and antiretroviral therapy (ART) toxicity monitoring are limited. Equations to estimate glomerular filtration rate (GFR) from serum creatinine were derived in Western populations and may be less accurate in this population.

**Methods:**

We compared results from published GFR estimating equations with a direct measure of GFR by iohexol clearance in 99 HIV-infected, ART-naïve Kenyan adults. Iohexol concentration was measured from dried blood spots on filter paper. The bias ratio (mean of the ratio of estimated to measured GFR) and accuracy (percentage of estimates within 30% of the measured GFR) were calculated.

**Results:**

The median age was 35 years, and 60% were women. The majority had asymptomatic HIV, with median CD4+ cell count of 355 cells/mm^3^. Median measured GFR was 115 mL/min/1.73 m^2^. Overall accuracy was highest for the Chronic Kidney Disease Epidemiology Consortium (CKD-EPI) equation. Consistent with a prior report, bias and accuracy were improved by eliminating the coefficient for black race (85% of estimates within 30% of measured GFR). Accuracy of all equations was poor in participants with GFR 60–90 mL/min/1.73 m^2^ (<65% of estimates within 30% of measured GFR), although this subgroup was too small to reach definitive conclusions.

**Conclusions:**

Overall accuracy was highest for the CKD-EPI equation. Eliminating the coefficient for race further improved performance. Future studies are needed to determine the most accurate GFR estimate for use in individuals with GFR <90 mL/min/1.73 m^2^, in whom accurate estimation of kidney function is important to guide drug dosing. Direct measurement of GFR by iohexol clearance using a filter paper based assay is feasible for research purposes in resource-limited settings, and could be used to develop more accurate GFR estimates in African populations.

## Introduction

HIV infection affects an estimated 34 million people worldwide, including more than 23 million individuals in sub-Saharan Africa. [1] Since the introduction of combination antiretroviral therapy (ART) in 1995, non-AIDS complications including chronic kidney disease (CKD) have emerged as leading causes of mortality and morbidity in high income countries. [2], [3] In resource-limited settings, where access to ART remains the priority, less is known about the prevalence and impact of comorbid conditions in HIV-infected individuals.

Both HIV and CKD disproportionately affect individuals of African descent. The classic kidney disease of HIV infection, HIV-associated nephropathy (HIVAN), is strongly linked to genetic polymorphisms found almost exclusively in individuals of African heritage. [4]–[6] HIVAN is the result of HIV infection and viral gene expression in the kidney, and ART has had a significant beneficial impact. [7] At the same time, ART has been associated with adverse effects on the kidney and with an increased prevalence of CKD and traditional CKD risk factors in Western populations. [8] [9] [10]

The impact of HIV infection and ART on the prevalence of CKD in Africa has important implications for global public health. [11] Although studies have suggested a high prevalence of decreased glomerular filtration rate (GFR) among HIV-infected individuals in Africa, there is substantial variability between different populations and between different methods of estimating GFR. [12] [13] [14] [15] [16]–[20] Available methods to estimate kidney function, including the Modification of Diet in Renal Disease (MDRD) and Chronic Kidney Disease Epidemiology Consortium (CKD-EPI) GFR estimating equations, [21] [22] and the Cockcroft-Gault creatinine clearance estimate, [23] were derived in Western populations and have not been well validated in African populations. A single study in 100 South African blacks suggested that performance of the MDRD and CKD-EPI equations was improved when the coefficient for race was eliminated; [24], [25] however, this modification has not been validated in other African populations. In addition, the study included only 20 individuals with HIV infection, which may also influence the relationship between serum creatinine, muscle mass, and GFR. Accurate estimation of kidney function is essential to determine the burden of CKD in the growing population of HIV-infected individuals in Africa, and to guide drug dosing and toxicity monitoring in this vulnerable population. In order to better understand the performance of available creatinine-based estimates of kidney function in African individuals with HIV, we compared these estimating equations to a direct measure of GFR in 99 HIV-infected Kenyan adults.

## Methods

### Ethics Statement

Participants provided written informed consent, and the protocol was approved by the MTRH Institutional Research and Ethics Committee (IREC) and by the Institutional Review Boards of Indiana University School of Medicine and the Mount Sinai School of Medicine.

### Study population

The Academic Model Providing Access to Healthcare (AMPATH) provides HIV care to more than 100,000 HIV-infected adults in western Kenya. Ambulatory, ART-naïve, HIV-infected adults aged 18–60 years were recruited from a single clinic at the Moi Teaching and Referral Hospital (MTRH) in Eldoret, Kenya. Pregnant and lactating women were excluded because of known limitations of creatinine-based estimates in pregnancy and limited data on the safety of iohexol during fetal development and infancy. Individuals with serum creatinine >1.6 mg/dL were excluded because of safety concerns related to use of the radiocontrast agent iohexol in the setting of CKD. A study coordinator pre-screened the charts of consecutive adult patients presenting for care and flagged the charts of potentially eligible patients. Providers referred interested patients to the study coordinator for detailed eligibility screening. Because women are overrepresented in the AMPATH population (>70%), [26] we oversampled men to ensure that we would have adequate numbers for analysis. In accordance with the approved protocol, no information was recorded on patients who were deemed ineligible or who declined to participate.

All participants completed a single study visit. Trained research staff performed a focused physical examination (weight, height, and blood pressure) and standardized interview to collect data on demographics (self-reported age, sex, and race), comorbid conditions, symptoms of HIV disease, and medication use, including prescription and over the counter medications and supplements. A peripheral intravenous catheter (IV) was inserted for serial blood draws, and a baseline blood sample was drawn for the measurement of CD4+ cell count (flow cytometry analyzed by MultiSET Four-Color Immunophenotyping Software, BD Biosciences), serum creatinine (Jaffe alkaline picrate reaction, Cobas Integra 400 chemistry analyzer, Roche Diagnostics), and hematocrit (Coulter Ac-T diff analyzer, Beckman Coulter, Brea, CA).

### Estimation of kidney function

GFR was estimated using the CKD-EPI and original 4-variable MDRD equations. [21] [22] [23] Based on the results of a South African study demonstrating improved performance without the race coefficient [24], [25], the equations were examined both with and without the respective coefficient for black race. Although Cockcroft-Gault creatinine clearance is not a true estimate of GFR, we also considered this kidney function estimate because it remains widely used for the purposes of drug dosing. [23] Cockcroft-Gault creatinine clearance was adjusted for body surface area (BSA) of 1.73 m^2^ to allow for comparison with other estimates and with measured GFR; for simplicity, all estimates of kidney function are referred to as estimated GFR (eGFR) in the results. All GFR estimates >200 mL/min/1.73 m^2^ were set to 200 mL/min/1.73 m^2^. This cutoff is consistent with the published literature and is based on the upper range of measured GFR in the CKD-EPI development cohort. [21]

### Direct measurement of GFR

The plasma disappearance of iohexol was used to measure GFR, as previously described. [27] The protocol for measurement of iohexol concentration was modified from a 2006 publication demonstrating excellent correlation between GFR measures obtained through conventional assays of venous plasma samples and measurements obtained using a filter paper-based assay. [28] A single dose of 5cc of iohexol (Omnipaque, GE Healthcare) was administered over 30 seconds through a butterfly needle inserted in the arm contralateral to the IV, followed by a 10cc normal saline flush. The syringe containing iohexol was weighed to the nearest 0.1 g prior to and immediately after injection to more accurately calculate the dose of iohexol administered. Blood was drawn from the IV at the time of insertion for an iohexol blank and at approximately 10, 30, 120, and 240 minutes after injection of iohexol, with exact times recorded. The protocol did not specify a later time point for participants with decreased GFR, as individuals with serum creatinine >1.6 mg/dL were not eligible for the study as described above. Participants were observed in the research unit throughout the study visit, and a cup of tea was provided before the last blood draw. Whole blood was spotted on pre-labeled filter paper at each time point and allowed to dry at room temperature. The 5 filter papers for each participant were placed in an individual sealed plastic bag containing a dessicant, and stored in a climate-controlled laboratory at MTRH (temperature 18–25C). Filter papers were shipped to the University of Rochester for the measurement of iohexol concentration by high performance liquid chromatography.

Dried blood spots were prepared according to a published protocol. [28] Briefly, a 6.3 mm diameter punch was taken from the center of the dried blood spot to create a disk containing 11.6 µL of blood. Disks were assayed in duplicate and the average value was reported. Any samples for which the duplicates differed by more than 20% were re-assayed; if the duplicates measured for the 120 and 240 minute samples differed by more than 20%, then all samples for that participant were re-assayed. If values <10 µg/mL were obtained, those time points were re-assayed by doubling the number of disks used. Because iohexol is not taken up by red blood cells, the plasma iohexol concentration was calculated from the whole blood concentration by correcting for the hematocrit [iohexol]/(1-hematocrit) as previously described. [28] Prior to performing the batched analysis of specimens for the current study, this assay was optimized in the University of Rochester laboratory using dried blood spots from individuals enrolled in a separate study, including simultaneously collected dried blood spots and plasma samples from 5 individuals. The coefficient of variation (CV) for the iohexol measurement was approximately 10% with the dried blood spots *vs* 1–3% with plasma samples. This variability is assumed to be random and should provide unbiased results when considering multiple time points. Niculescu *et al*. previously demonstrated good agreement between iohexol GFR measurements using dried blood spots *versus* serum (R^2^ 0.95) [28].

The two-compartment plasma disappearance of iohexol was calculated using the following equation: I/(expA/α + expB/β) X 1.73/BSA, where I is dose of the iohexol (mg), expA is the intercept of the slow curve and α is its corresponding slope, expB is the intercept of the fast curve and β is its corresponding slope. The fast and slow curves were determined from concentrations at 10 and 30 minutes and from concentrations at 120 and 240 minutes, respectively. The single-compartment plasma disappearance of iohexol was also calculated from the slow curve as previously described. [29] For cases in which the 4-point GFR could not be calculated because of missing or invalid results at 10 or 30 minutes, the 2-point single-compartment GFR was accepted after review by a single investigator (GS) blinded to the eGFR results. Measured GFR was corrected to 1.73 m^2^ body surface area (BSA). To allow for direct comparison of eGFR and iGFR results, values >200 mL/min/1.73 m^2^ were set to 200 mL/min/1.73 m^2^.

### Statistical Analysis

The agreement between eGFR and GFR measured by iohexol clearance (iGFR) was first examined using Bland Altman analysis. This analysis was performed on the natural log scale as is common practice with GFR measures. The resulting metrics of agreement are on a relative scale after transformation back to the natural scale. Bland Altman plots were created after back transformation by plotting the geometric mean of eGFR and iGFR on the x-axis and the ratio of eGFR: iGFR on the y-axis. The ratio, rather than the difference, reflects the greater clinical relevance of small absolute differences at lower GFR.

The Bland Altman analysis provides three metrics of agreement: the bias ratio, which is informative for determining the distance between the two measures at the central tendency; the variance inflation, which quantifies the shrinking or expanding of the distribution of one measure relative to the other; and the correlation, which assesses the degree to which the ordering of values is maintained between the two measures. The bias ratio, or mean of the ratio of eGFR: iGFR, was interpreted as indicating overestimation of the iGFR when values were >1 and underestimation of iGFR when values were <1. The variance inflation, or ratio of the standard deviation of eGFR: standard deviation of iGFR, was interpreted as indicating greater variability in the GFR estimates compared to the measured GFRs when the value was >1 and less variability in the GFR estimates compared to the measured GFRs when the value was <1. In addition to the Bland Altman analysis, the accuracy of each equation was calculated as the % of estimates within 10% and 30% of the iGFR.

In order to build a preliminary prediction model for GFR in the dataset, the relationship between iGFR and serum creatinine was assessed to determine the shape of the function. Other variables considered in the multivariable models were age, sex, height, weight, body mass index (BMI), CD4 (dichotomized at 200 cells/mm^3^), and all interactions between these factors and serum creatinine. The longitudinal GFR estimating equations were of the form log(iGFR) =  α + βlog(X) + γ Z + ε, where X represented the serum creatinine values (natural log transformed), centered at 0.7 mg/dL, and Z represented all other continuous and categorical factors. Exponentiating the equation yielded the following parameter estimates: the intercept, α, was the average iGFR in the training data, β was the power that quantified the impact of the serum creatinine values as they deviated from the centered value, and γ was the multiplier that described the impact of a categorical factor. For example, an individual with serum creatinine twice that of another would be expected to have 2^β^ the GFR. Similarly, an individual with CD4<200 cells/mm^3^ would be expected to have γ^1^ times the GFR of an individual with CD4≥200 cells/mm^3^. The quantity, ε, represented the error between eGFR and iGFR. The full model was the model with the highest Akaike information criterion (AIC) among all nested models. A simplified version using only significant predictors at the alpha = 0.01 level was assessed for comparison. Separate sensitivity analyses were repeated after excluding participants on trimethoprim-sulfamethoxazole, which is known to interfere with the tubular secretion of creatinine, [8] and after excluding participants in whom the 2-point iGFR was used.

## Results

A total of 100 HIV-positive black adults were enrolled. One male participant with serum creatinine 0.8 mg/dL was excluded from analysis because of a missing hematocrit, which is required to calculate plasma iohexol concentration. The median age of included participants was 35 years (range 22–57 years), and 60% were women (Table 1). Twelve participants had a BMI ≤18.5, and no participant had a BMI of 30 or greater (range 16–27). There were no participants with documented diabetes mellitus, and a single female participant reported the use of antihypertensive medications. The majority of participants had asymptomatic HIV, and approximately half had a CD4 cell count >350 cells/mm^3^.

**Table 1:** Clinical characteristics of included participants (n = 99).

	Median (IQR) or n (%)
Age, years	35 (31–40)
Female sex	60 (61%)
Weight, kg	60 (53–65)
Height, m	1.7 (1.6–1.7)
Body mass index	22 (20–24)
Body surface area, m^2^	1.7 (1.6–1.7)
CD4 cell count, cells/mm^3^	355 (219–445)
WHO stage	
1	74 (75%)
2	14 (14%)
3	10 (10%)
4	1 (1%)
Serum creatinine, mg/dL	0.70 (0.64–0.80)
Iohexol GFR, mL/min/1.73m^2^	115 (102–137)
CKD-EPI eGFR, mL/min/1.73m^2^	
4-variable equation	130 (120–138)
Without coefficient for black race	113 (104–120)
MDRD eGFR, mL/min/1.73m^2^	
4-variable equation	130 (117–155)
Without coefficient for black race	108 (97–128)
Cockcroft-Gault CrCl, mL/min/1.73m^2^	106 (96–124)

IQR, interquartile range; WHO, World Health Organization Stage of HIV disease; GFR, glomerular filtration rate; eGFR, estimated GFR; CKD-EPI eGFR, eGFR calculated using the Chronic Kidney Disease Epidemiology Collaboration equation; MDRD eGFR, estimated glomerular filtration rate (eGFR) calculated using the Modification of Diet in Renal Disease equation; CrCl, creatinine clearance. Comorbid disease was rare; no participant had diabetes mellitus or obesity, and a single participant reported the use of antihypertensive medications.

**Figure 1:**
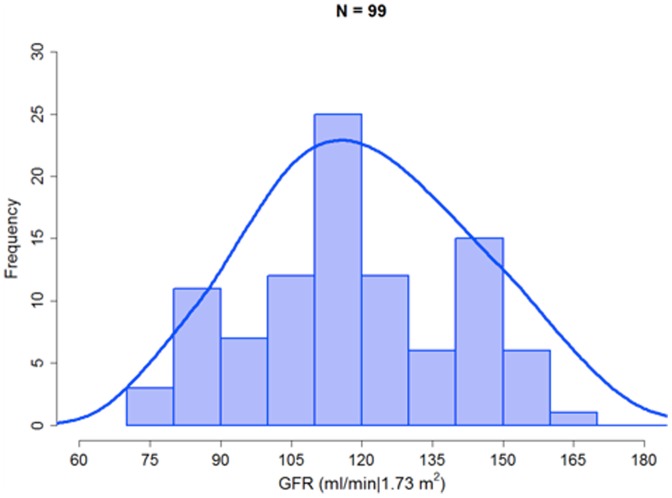
Distribution of glomerular filtration rate (GFR) measured by iohexol clearance in 99 HIV-infected study participants.

The median serum creatinine was 0.7 (range 0.4–1.1 mg/dL), and median eGFR ranged from 106 mL/min/1.73 m^2^ for Cockcroft-Gault to 130 mL/min/1.73 m^2^ for the 4-variable MDRD and CKD-EPI estimates. GFR estimates >200 mL/min/1.73 m^2^ were set to 200 mL/min/1.73 m^2^ in 8 male participants aged ≤35 years. Median iGFR was 115 ml/min/1.73 m^2^, and 85 participants had an iGFR ≥90 ml/min/1.73 m^2^ (Figure 1). The iGFR was set at 200 mL/min/1.73 m^2^ in a single female participant with a serum creatinine of 0.5 mg/dL and an iGFR of 212 mL/min/1.73 m^2^. The 2-point iGFR was substituted for the 4-point iGFR in 16 participants with missing or implausible results at 10 or 30 minutes. Among participants with results available for both, the correlation between 2-point and 4-point iGFR was excellent (R^2^ 0.91).

**Figure 2:**
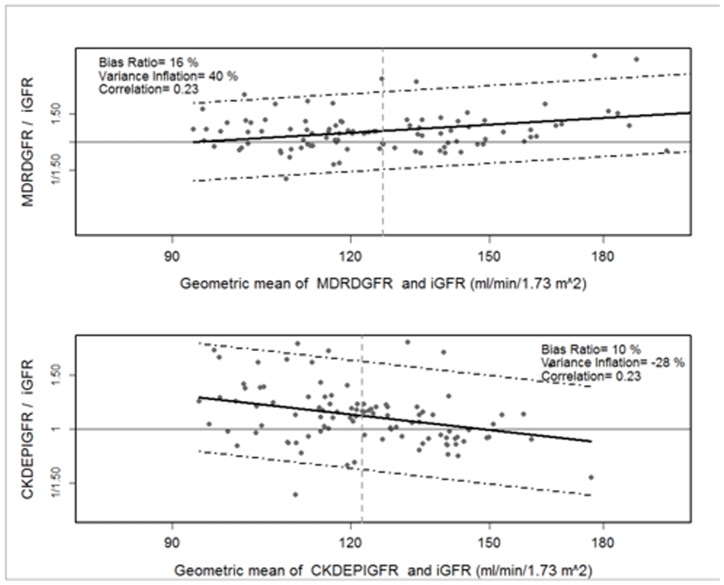
Bland Altman plots showing the agreement between measured and estimated glomerular filtration rate. iGFR, glomerular filtration rate measured by iohexol clearance. MDRD, 4-variable Modification of Diet in Renal Disease equation; CKD-EPI, Chronic Kidney Disease Epidemiology Collaboration equation.

**Figure 3:**
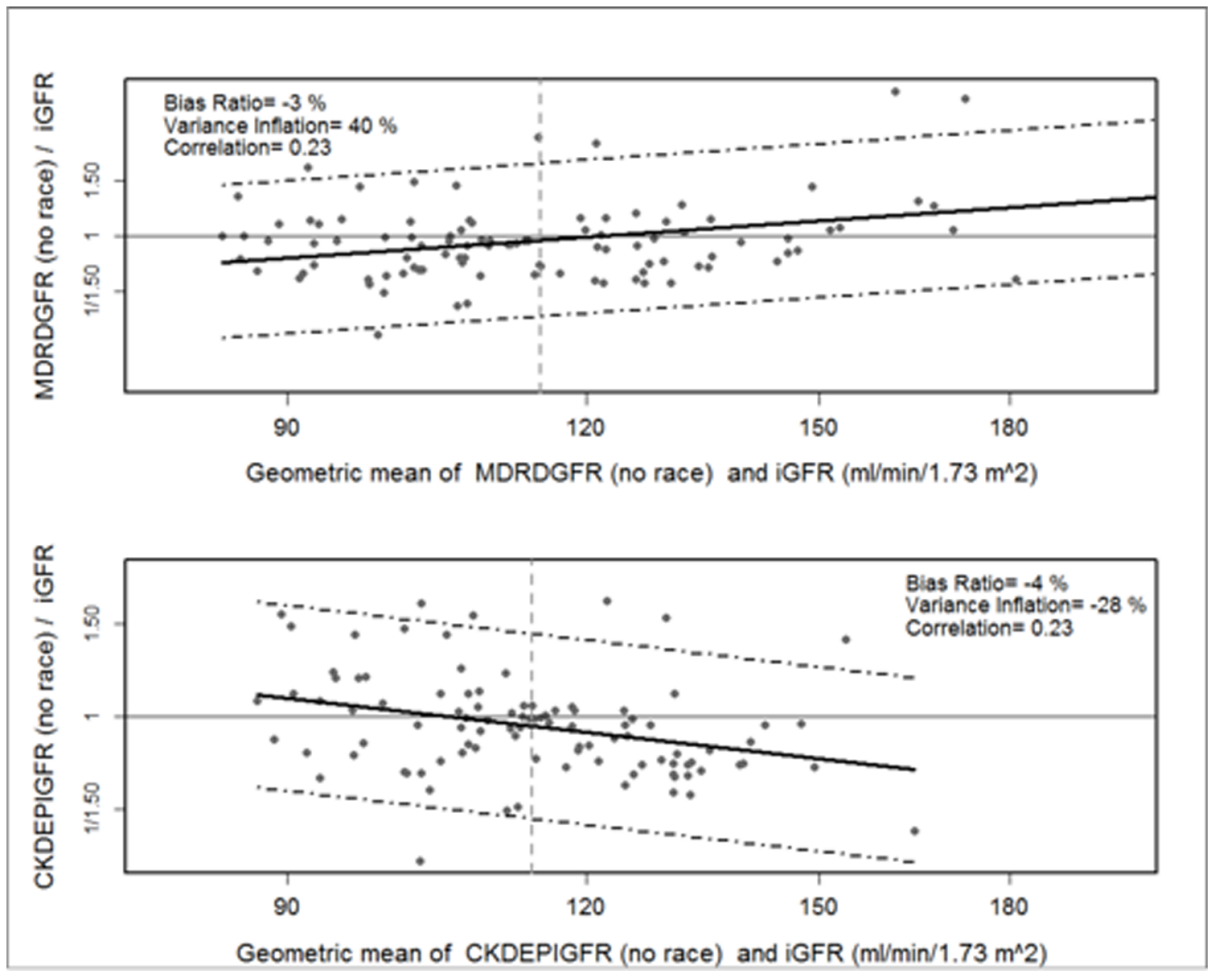
Bland Altman plots showing the agreement between measured and estimated glomerular filtration rate after elimination of the coefficient for black race. iGFR, glomerular filtration rate measured by iohexol clearance. MDRD, 4-variable Modification of Diet in Renal Disease equation; CKD-EPI, Chronic Kidney Disease Epidemiology Collaboration equation.

As compared to the direct measure of GFR by iohexol clearance, the CKD-EPI equation had the best performance overall (Figure 2, Table 2). Elimination of the race coefficient improved the bias and accuracy of the CKD-EPI and MDRD estimates, although the differences were small (Figure 3, Table 2). For all equations, the correlation between eGFR and iGFR was poor (R^2^ ≤0.23), while the accuracy within 30% of iGFR was moderate (73–85%). When participants were stratified by iGFR, accuracy within 30% of iGFR was high for all equations in participants with GFR ≥90 mL/min/1.73 m^2^ (80–92% of estimates within 30% of iGFR). In the small subgroup of participants with GFR 60–89 ml/min/1.73 m^2^ (n = 14), accuracy was poor for all equations. Accuracy of the MDRD and CKD-EPI equations remained low in this subgroup when the race coefficient was eliminated (50% of estimates within 30% of iGFR). Although overall accuracy of the Cockcroft-Gault equation was poor, this estimate had the highest accuracy in participants with iGFR 60–89 ml/min/1.73 m^2^ (64% within 30% of iGFR). Accuracy within 10% of iGFR was poor for all equations. Bias was minimal in the setting of GFR ≥90 mL/min/1.73 m^2^, regardless of the equation. In participants with iGFR 60–89 mL/min/1.73 m^2^, bias was more substantial and all equations overestimated iGFR. In separate sensitivity analyses, results were qualitatively similar after excluding 3 participants on trimethoprim-sulfamethoxazole (including the only participant with WHO stage 4 HIV disease), and after excluding 16 participants in whom the 2-point iGFR was used as the direct measure of GFR (data not shown).

**Table 2:** Performance of creatinine-based estimates of glomerular filtration rate (GFR).

	Overall (N = 99)	mGFR≤90 (N = 14)	mGFR>90 (N = 85)
**Accuracy**			
*% of estimates within 30% of iGFR*			
MDRD	73%	21%	81%
MDRD without adjustment for race	83%	50%	88%
CKD-EPI	82%	21%	92%
CKD-EPI without adjustment for race	85%	50%	91%
Cockcroft-Gault, BSA-adjusted	78%	64%	80%
*% of estimates within 10% of iGFR*			
MDRD	26%	0%	31%
MDRD without adjustment for race	34%	36%	34%
CKD-EPI	32%	0%	38%
CKD-EPI without adjustment for race	35%	14%	39%
Cockcroft-Gault, BSA adjusted	31%	29%	32%
**Bias Ratio** (mean(eGFR/iGFR))			
MDRD	***1.18***	***1.58***	***1.12***
MDRD without coefficient for race	***0.97***	***1.30***	***0.93***
CKD-EPI	***1.10***	***1.51***	***1.05***
CKD-EPI without coefficient for race	***0.96***	***1.31***	***0.91***
Cockcroft-Gault, BSA-adjusted	0.94	***1.24***	***0.90***
**Variance Inflation** (SD(eGFR)/SD(iGFR))			
MDRD	***1.40***	***3.27***	***1.83***
MDRD without coefficient for race	***1.40***	***3.27***	***1.83***
CKD-EPI	***0.72***	1.78	0.95
CKD-EPI without coefficient for race	***0.72***	1.77	0.94
Cockcroft-Gault, BSA-adjusted	***1.41***	***2.78***	***1.87***

iGFR, glomerular filtration rate measured by iohexol clearance; eGFR, estimated glomerular filtration rate; MDRD, 4-variable Modification of Diet in Renal Disease equation; CKD-EPI, Chronic Kidney Disease Epidemiology Collaboration equation; BSA-adjusted, corrected for average body surface area of 1.73 m^2^ (expressed in mL/min/1.73 m^2^).

***Bold*** signifies a bias ratio or variance inflation ratio significantly different from 1.0 (0%).

We sought to build a preliminary prediction model for the estimation of iGFR from serum creatinine and patient characteristics. Because of the relatively small sample size, the model was derived using the full dataset (Table 3). Variables that contributed to the final model included serum creatinine, age, sex, height, and CD4 (dichotomized at 200 cells/mm^3^). Weight and BMI were not significant predictors of GFR in our population (p>0.05) and were not included in the final model. The final prediction model had high accuracy in our population (92% of estimates within 30% of measured GFR), but failed to explain a large proportion of the variability in GFR (R^2^ 18%). Parameter estimates were similar excluding participants on trimethoprim-sulfamethoxazole (data not shown). In a separate sensitivity analysis, excluding participants in whom the 2-point iGFR was used as the direct measure of GFR did not change the variables included in the final model (data not shown).

**Table 3:** Estimating equations for prediction of GFR from serum creatinine, developed using iohexol GFR data from 99 HIV-infected individuals.

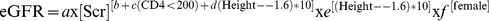										
Model	*a*	*b*	*c*	*d*	*e*	*f*	R^2^	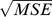	% within 30%	% within 10%
None	116.4 ±2.3	0	0	0	0	0	–	0.199	84%	36%
Full	127.2 ±5.6	−0.393 ±0.136	−0.485 ±0.280	0.302 ±0.122	0.943 ±0.030	0.908 ±0.041	18%	0.184	92%	44%
Simple	116.5 ±2.3	−0.182 ±0.091	0	0	0	0	4%	0.196	85%	44%

eGFR, estimated glomerular filtration rate; SCr, serum creatinine in mg/dL; height in meters; 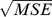, root mean square error calculated based on the log transformed iohexol GFR.

Because height was an important predictor of iGFR in our dataset, we also considered the performance of the pediatric Schwartz formula, which estimates GFR from serum creatinine and height [GFR in mL/min/1.73 m^2^  =  (constant × height in cm)/creatinine in mg/dL]. [30] Using published constants for adolescents, [31] the Schwartz formula significantly overestimated iGFR, while using a more recently derived constant tended to underestimate iGFR in our population [32]. A constant of 0.49 minimized the bias of this estimate in our population (bias ratio 0.99), although accuracy was lower than that of the full model or the CKD-EPI equation (80% of estimates within 30% of iGFR).

## Discussion

In this study of ART-naïve Kenyan adults with largely asymptomatic HIV infection, available GFR estimates were reasonably accurate in participants with normal or high GFR. Although the correlation between measured and estimated GFR was poor, a more clinically relevant measure of accuracy within 30% of measured GFR was high for all equations in participants with GFR ≥90 mL/min/1.73 m^2^ (80–92% of estimates within 30% of iGFR). Overall, accuracy was highest for the CKD-EPI equation, consistent with the performance of this estimate in Western populations both with and without HIV. [22] [33] In contrast to Western populations where creatinine-based GFR estimates including MDRD and CKD-EPI are more accurate in the setting of decreased GFR, the accuracy of all equations was poor in the small subgroup of participants with GFR <90 mL/min/1.73 m^2^. Studies including a larger number of African participants with decreased GFR are needed to confirm this finding and to determine the most appropriate equation for use in that setting.

As previously suggested in a study of South African blacks [24], bias and accuracy of the CKD-EPI and MDRD equations were improved by eliminating the coefficient for race. This finding and the poor performance of GFR estimates in the small subgroup of participants with decreased GFR suggest that the relationship between serum creatinine, diet, muscle mass, and GFR in African blacks is different than that observed in African-Americans included in the CKD-EPI and MDRD development cohorts. It is also interesting that weight was not a significant predictor of iGFR in our population, consistent with the poor performance of the Cockcroft-Gault equation, while height remained a significant predictor. Although the results of our prediction model should be validated in other African populations and in larger studies, there is a precedent for the inclusion of height rather than weight in the pediatric Schwartz formula [30]. A modified version of the Schwartz formula using a constant of 0.49 provided minimal bias and reasonable accuracy in our population, suggesting that this simple bedside GFR estimate could be studied as an alternative to more complex equations in settings without access to automated GFR reporting.

The results of the current study suggest a need for larger studies in diverse African populations to determine the most accurate GFR estimate for use in research and clinical care. Although the subgroup of participants with decreased GFR was too small to draw firm conclusions, all of the available GFR estimating equations tended to overestimate GFR in the small subgroup of participants with GFR 60–89 mL/min/1.73 m^2^. Because estimates of kidney function are most likely to influence prevalence estimates and clinical decision-making in individuals with decreased GFR, we have included the results from this small subgroup to reinforce the need for additional research and to caution clinicians to use clinical judgment when evaluating GFR estimates. The current study was not intended to describe the true population distribution of GFR; a previous cross-sectional study estimated that approximately 5% of ART-naïve adults in the AMPATH clinic population have an eGFR <60 mL/min/1.73 m^2^ as estimated by the 4-variable MDRD equation [12]. If future studies confirm our results in individuals with GFR <90 mL/min/1.73 m^2^ and extend them to individuals with GFR <60 mL/min/1.73 m^2^, the observed overestimation of GFR would have significant implications for ART regimen selection, dosing, and toxicity monitoring.

The current study also opens the door for future studies to determine the most accurate GFR estimate for use in African populations, demonstrating the feasibility of direct GFR measurement for research purposes in resource-limited settings. The plasma clearance of iohexol was described as a simple alternative to inulin clearance for the direct measurement of GFR in 1995. [27] Since then, iohexol clearance has been widely adopted as a gold standard measure of GFR in epidemiologic studies, and as an alternative to more cumbersome and expensive radionuclide measurements [22] [32]. [34] Unlike other techniques for direct GFR measurement, iohexol clearance does not require a nuclear medicine facility. The innovative application of filter paper technology, which eliminates the need for cumbersome specimen processing and specialized laboratory facilities for HPLC, makes this approach even more appealing for use as a research tool in resource-limited settings. The potential to obtain blood by finger prick [28] and the strong correlation between 4-point and 2-point iGFR measures may allow for further simplification of the iohexol clearance protocol in future studies.

Although this is the largest study to evaluate the performance of GFR estimates in African individuals with HIV infection, our findings should be interpreted in light of several limitations. First, the measurement of iohexol concentration using dried blood spots may have introduced additional variability into our “gold standard” measure. Although variability of the iohexol assay at an individual time point is greater with dried blood spots than with serum (CV 10% *versus* 1–3%), this variability is assumed to be random and should therefore provide unbiased results when considering multiple time points. In addition, the dried blood spots were assayed in duplicate, and any samples for which the duplicates differed by more than 20% were re-assayed. Niculescu *et al*. previously demonstrated good agreement between iohexol GFR measurements using dried blood spots *versus* serum (R^2^ 0.95), concluding that this approach was appropriate for use in epidemiologic studies [28]. Nonetheless, we acknowledge the potential for increased variability in our gold standard measure, and have focused our discussion on large, clinically relevant discrepancies of >30% between measured and estimated GFR.

A second important limitation is the small number of participants with mildly decreased kidney function and the exclusion of individuals with moderately decreased kidney function. Because of IREC concerns about the safety of iohexol in individuals with CKD, the current study included a small number of individuals with GFR <90 mL/min/1.73 m^2^, and no participants with a GFR <60 mL/min/1.73 m^2^. In clinical practice, accurate estimation of GFR is most important in individuals with decreased GFR, as dose adjustment of tenofovir and most nucleoside reverse transcriptase inhibitors is recommended at GFR <50 mL/min/1.73 m^2^. More recently, iohexol clearance has been used safely to measure GFR in individuals with CKD, [22] [32] [34] and future studies should include a larger number of participants with mild to moderately decreased GFR.

Third, our sample size was too small to allow for the internal validation of a new equation to estimate GFR or for the exploration of possible interactions, and the preliminary prediction model failed to explain a large proportion of the variability in GFR. The CKD-EPI equation, but not the MDRD or Cockcroft-Gault equations, allows the relationship between sex and GFR to vary by the level of serum creatinine; existing equations do not include any other interaction terms. [22] Despite the preliminary nature of the model and the need for validation in other populations and in individuals with decreased GFR, our results suggest that future efforts to develop a more accurate GFR estimate in African populations should consider clinical and demographic parameters in addition to those included in existing equations.

Fourth, serum creatinine was measured by Jaffe reaction rather than using an enzymatic assay traceable to isotope dilution mass spectrometry (IDMS) reference standards, which is known to be more accurate at low levels and to improve the performance of the MDRD and CKD-EPI equations. The original version of the MDRD equation used in the current study is intended for use with non-standardized creatinine values; [21] however, it is likely that performance of the CKD-EPI equation would be improved if an IDMS-traceable assay were used. Nonetheless, the non-standardized Jaffe reaction is still widely used for the measurement of serum creatinine in resource-limited settings throughout Africa, increasing the applicability of our findings. Similarly, we did not measure cystatin C, which may be combined with serum creatinine to improve the accuracy of GFR estimates, [35] [33] because the routine measurement of cystatin C is not feasible in resource-limited settings.

Finally, the results of the current study may not be generalizable to other African populations, to ART-treated individuals, or to patients with more advanced HIV disease. In a previous study of >49,000 non-pregnant adults newly enrolled in the AMPATH HIV program, the mean age and weight were similar to our study population. [26] Approximately 60% of patients presenting for care had an indication for the immediate initiation of ART according to local guidelines at the time (CD4 cell count <200 cells/m^3^ or WHO stage 3–4 disease). Because we felt that it would be unethical to delay the initiation of ART to allow participation in the current study, the majority of our participants had asymptomatic HIV disease. Nonetheless, nearly half of our study population had a CD4 cell count <350 cells/m^3^ and would be considered candidates for ART initiation according to the current World Health Organization criteria. [36].

In this study of 99 ambulatory ART-naïve adults in Kenya, we have demonstrated both the feasibility and the need for larger studies to determine the most accurate estimate of kidney function in HIV-infected individuals in Africa. With expanding access to ART and limited resources for toxicity monitoring and management in most African nations, accurate estimation of kidney function is important to guide drug dosing and to identify populations at increased risk for drug toxicity. The protocol for iohexol GFR using filter paper technology provides a strong epidemiologic tool to investigate the burden of HIV-related kidney disease in this and other resource-limited settings.
